# Lung microbial dysregulation and TNF inhibition are associated with worsened nontuberculous mycobacterial lung disease

**DOI:** 10.3389/fmicb.2026.1923035

**Published:** 2026-09-15

**Authors:** Ethan G. Napier, Isaac R. Cinco, Delphine C. Malherbe, Ethan V. Stuart, Michael H. Davies, Caroline N. Leach, Evan Damron, Mahmut Gokmen, Amy Leestemaker-Palmer, Stephanie Nuss, Cody Bumgardner, Steven G. Kohama, Luiz Bermudez, Kevin L. Winthrop, Cristina Fuss, Eliot R. Spindel, Ilhem Messaoudi

**Affiliations:** 1 Department of Microbiology, Immunology, and Molecular Genetics, College of Medicine, University of Kentucky, Lexington, KY, United States; 2 Division of Neuroscience, Oregon National Primate Research Center, Oregon Health and Science University, Beaverton, OR, United States; 3 Department of Pathology and Laboratory Medicine, College of Medicine, University of Kentucky, Lexington, KY, United States; 4 Department of Microbiology, College of Sciences, Oregon State University, Corvallis, OR, United States; 5 Division of Infectious Diseases, Schools of Medicine and Public Health, Oregon Health and Sciences University, Portland, OR, United States; 6 Department of Radiology and Biomedical Imaging, School of Medicine, Yale University, New Haven, CT, United States

**Keywords:** lung, microbiome, nontuberculous mycobacteria, rhesus macaque, TNF

## Abstract

**Introduction:**

Nontuberculous mycobacteria (NTM) are ubiquitous bacteria that cause a spectrum of diseases, most notably pulmonary disease (NTMPD). The host factors contributing to the heightened susceptibility and severity of NTMPD in elderly individuals are poorly understood. Prior studies have reported increased incidence of NTMPD in individuals receiving immune modulatory biologics such as anti-TNF and JAK-STAT inhibitors. Moreover, we recently described that age-related changes in the lung microbiome, notably the loss of a main commensal *Tropheryma* species, may contribute to increased severity. Therefore, in this study we explore the hypothesis that TNF-inhibition and a disrupted lung microbiome are key factors associated with poor NTMPD outcomes.

**Methods:**

Young (4–7 years old) rhesus macaques were either pretreated with: (1) nebulized amikacin and vancomycin to disrupt the lung microbiome; (2) the TNF inhibitor Inflectra to suppress Th1 responses; or (3) left untreated. Animals were subsequently inoculated with *M. avium* subsp. *hominissuis* (MAH) in the right lung. Bacterial load, radiographic changes, immune responses, and microbiome composition were monitored longitudinally.

**Results and discussion:**

Antibiotic-treated animals experienced significant microbiome compositional shifts including the depletion of *Tropheryma* from the lung microbiome. One antibiotic-treated animal developed and resolved cavitary disease after the lung microbiome returned to homeostasis. One Inflectra-treated animal developed chronic granulomatous disease. No control animals showed granulomas. These data suggest that lung microbiome disruption and TNF inhibition are associated with increased susceptibility to NTM granulomatous disease.

## Introduction

Nontuberculous mycobacteria (NTM) are ubiquitous microbes that can infect human lungs via aerosolized particles (Honda et al., 2015; Jeon, 2019). Individuals with a compromised immune system or underlying lung conditions are at risk of developing NTM pulmonary disease (NTMPD) or disseminated NTM disease (Loebinger et al., 2023; Nqwata and Ouédrago, 2022). NTM disease first gained clinical relevance during the HIV/AIDS epidemic. These patients lacked a strong Th1 response due to the depletion of their CD4+ T cells, which allowed for disseminated NTM disease (Hill, 1991). In contrast, most HIV-negative NTM cases are pulmonary. Risk factors for NTM infection can include pharmacological immunosuppression such as treatment with tumor necrosis factor-alpha (TNF) antagonists, corticosteroids, and JAK-STAT inhibitors (Winthrop et al., 2009; Andréjak et al., 2013; Anand et al., 2020). Additional risk factors for NTMPD also include underlying lung conditions including cystic fibrosis (CF), chronic obstructive pulmonary disease (COPD) and bronchiectasis. Age is another major risk factor, and it is notable that most fatal NTM cases occur in people > 65 (Dhasmana et al., 2024; Harada et al., 2025). Aging results in immunosenescence characterized by reduced circulating naïve T cells, increased memory T cells and chronic low-grade inflammation (Lee et al., 2022; Liu et al., 2023; Oh et al., 2019; Wrona et al., 2024). Additionally, aging dysregulates the lung microbiome, which can impact the prevalence and severity of respiratory diseases (Lee et al., 2019; Napier et al., 2025; Rhoades et al., 2022).

These observations indicate that impaired Th1 immune response and a dysregulated lung microbiome may be underlying mechanisms for the increased susceptibility and severity of NTM disease in the aged population (McGarvey and Bermudez, 2002; Winthrop et al., 2013; Winthrop et al., 2008). However, these mechanisms are poorly understood due to the limitations with rodent models of infection and human clinical studies. NTM infections in rodents can result in a disseminated rather than pulmonary disease (Cha et al., 2015), and their specific-pathogen-free status prohibits translatable microbiome signatures (Boukhvalova et al., 2007; Liu and Kimura, 2007; Toapanta and Ross, 2009; Wong et al., 2014; Yangco et al., 1989). Clinical studies face inherent challenges in sample acquisition and confounding variables like smoking history, diet, and comorbidities. We developed a rhesus macaque model that overcomes these limitations and recapitulates key hallmarks of human NTMPD including clinical and radiologic features, as well as immune response to infection (Winthrop et al., 2016). Rhesus macaques are a widely accepted model for the impact of immunosenescence in respiratory infections (Rivera et al., 2019; Speranza et al., 2022). Using this model, we previously demonstrated increased disease severity upon NTM infection with *M. avium* subsp. *hominissuis* strain 101 (MAH) in aged animals as measured by persistent radiographic findings consistent with bronchiectasis as well as presence of pulmonary lesions at necropsy, increased immune cell infiltration and activation (Cinco et al., 2024; Napier et al., 2025).

These prior studies highlighted that while young animals generated a predominantly Th1 immune response (with high levels of IFNγ, IL-12, TNFα, and CD40L), aged animals generated predominantly an acute phase response indicated by high levels of IL-6 and had prolonged elevation of the chemoattractant CCL2 (Napier et al., 2025). Moreover, the loss of an uncultured Tropheryma species in aged animals and the negative correlation between its abundance and the severity of radiographic findings suggested that the lung microbiome plays a protective role against MAH disease (Cinco et al., 2024; Napier et al., 2025). These findings suggest that a Th1 response and Tropheryma abundance can modulate NTMPD severity. To test this hypothesis, we pretreated a cohort of rhesus macaques with either antibiotics to deplete the lung microbiome or Inflectra to inhibit TNF responses. Infection was followed for 310 days with longitudinal CT imaging, microbiome profiling, immune cell characterization, histology and proteomic analysis. One Inflectra-treated animal developed pulmonary granulomatous disease, and one antibiotic-treated animal developed and resolved cavitary disease. Moreover, antibiotic- and Inflectra-treated animals showed significant dysregulation of immune mediators and immune cell infiltration compared to the control animals indicating that TNF signaling and microbiome composition of the lung microbiome can impact the outcome of MAH infection.

## Materials and methods

### Ethics statement

Animal work was performed in accordance with the recommendations described in the Guide for the Care and Use of Laboratory Animals of the National Institute of Health, the Office of Animal Welfare and the Animal Welfare Act, United States Department of Agriculture. The studies were approved by the Institutional Animal Care and Use Committees (IACUC) at the Oregon National Primate Research Center.

### Cohort description, animal infection, and sample collection

Twelve young (4–7 years; 6 females, 6 males) colony-bred Indian-origin rhesus macaques (*Macaca mulatta*) were randomly assigned into three sex-matched groups (Figure 1): control, pre-treatment with 15 mg/kg amikacin and 4 mg/kg vancomycin delivered via nebulization at 3, 2, and 1 day prior to infection (antibiotic-treated), as well as pre-treatment with 5 mg/kg Inflectra delivered intravenously 7, 5 and 1 week prior to infection followed by treatment with Inflectra 49, 105, and 161 DPI (Inflectra-treated) (Supplementary Table 1). Following pre-treatment, animals were intrabronchially inoculated in the right upper, middle, and caudal lobe with a total of 6.8 * 10^8^ CFU of *M. avium* subsp. *hominissuis* strain 101 (MAH) divided equally between the 3 sites (Cinco et al., 2024). The right accessory lobe that macaques have was not inoculated with MAH. Thoracic CT, bronchoalveolar lavage fluid (BALF), oral, nasal, and fecal swabs were collected throughout the study (Figure 1).

**FIGURE 1:**
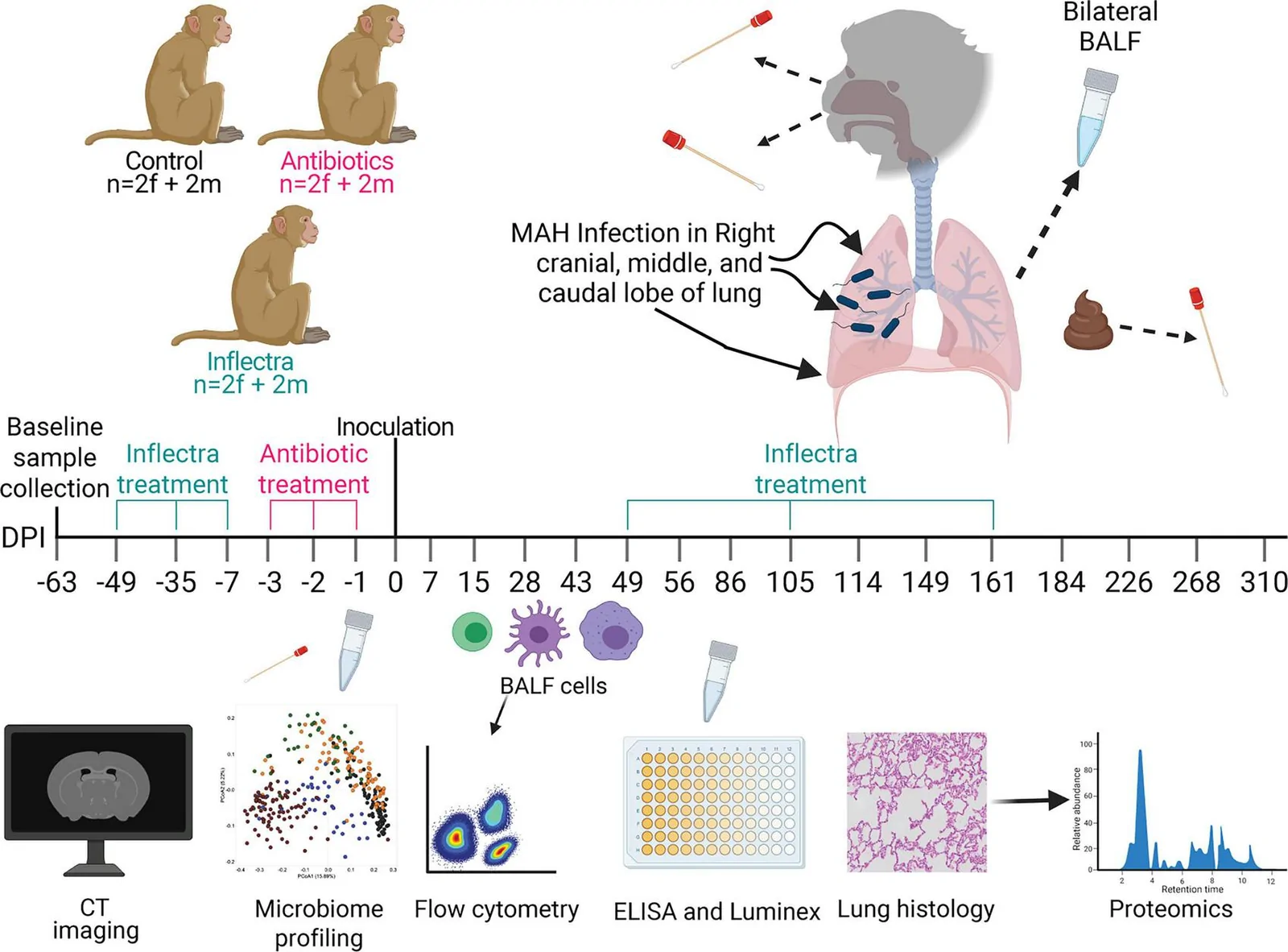
Experimental design. 12 young adult (4–7 years; 6 female and 6 male) rhesus macaques were segregated into 3 groups of 4: [1] Control, [2] antibiotic (Amikacin/Vancomycin) administration, and [3] Inflectra administration. Animals were inoculated in the right cranial, middle, and caudal lobe with a bolus of 6.8 × 10^8^ CFU of MAH split between the three sites. Nasal, oral, fecal, and bilateral bronchoalveolar lavage fluid (BALF) samples were collected at the indicated days post inoculation (DPI). BALF was probed via microbiome profiling, flow cytometry, ELISA, and Luminex assay. Microbiome profiling was done on the nasal, oral, and fecal swabs. Computed tomography (CT) scans were acquired, and severity scores were determined 0, 12, 40, 83, 110, 145, 181, 222, 264, and 299 DPI. Lung histology was done at necropsy and proteomics data were acquired from the tissues.

### CT severity scoring system

CT scans were qualitatively evaluated by a blinded radiologist per our published method (Cinco et al., 2024) and assigned a severity score based on appearance of ground glass, mucus, volume loss, lymphadenopathy, detection and severity of bronchiectasis, presence and size of cavitations, scarring, and tree in bud opacity with a score of [1] being minimal findings to [6] being severe.

### Bacterial culture and load

Bacterial load was determined by [1] plating serial dilutions of the BALF supernatant onto Lowenstein-Jensen agar plates and enumerating the number of colony-forming units (CFU) after 8 weeks of incubation at 37°C/5% CO_2_ and [2] performing real-time quantitative PCR (qPCR). DNA was extracted from BALF cells using the DNeasy PowerSoil Pro Kit (Qiagen, Germantown, MD) according to the manufacturer’s instructions. Bacterial burden of MAH at 0, 7, 16, 28, and 44 DPI was determined by qPCR using SsoAdvanced Universal SYBR Green Supermix (Bio-Rad, Hercules, CA) and primers specific for the IS1311 insertion sequence found in *M. avium* (Whittington et al., 1998). Each run was initiated at 50°C for 2 min, then 95°C for 10 min, followed by 40 cycles at 95°C for 15 sec then 60°C for 1 min using a QuantStudio 3 Real-Time PCR System (Thermo Fisher Scientific). Extracted MAH DNA was used as the quantification standard.

### Flow cytometry

1 * 10^6^ BALF cells were incubated with Ghost Dye Violet 510 (Tonbo Biosciences, San Diego, CA), then surface stained with antibodies against CD20, CD27, IgD, CD4 (Biolegend, San Diego, CA), CCR7 (BD Biosciences, Franklin Lakes, NJ), CD8b (Beckman Coulter, Brea, CA), and CD28 (Tonbo Biosciences, San Diego, CA). Cells were then fixed and permeabilized before the addition of anti-Ki67 (BD biosciences, Franklin Lakes, NJ) to assess proliferation. This panel allows for the identification of B cells (CD20) and T cells (CD8b, CD4) as well as naïve and memory subsets as previously described (Rhoades et al., 2022). Another 10^6^ BALF cells were stained with CD206, CD14, HLA-DR, CD8a, CD123, CD16 (Biolegend, San Diego, CA), CD11c (Invitrogen, Waltham, MA), and Granzyme-B (BD Biosciences) to assess frequency of innate immune cells (Rhoades et al., 2022). To measure frequency of antigen-specific T cells, monocyte-derived macrophages, and DCs after MAH infection, 10^6^ BALF cells were stimulated with 1 mg/mL MAH lysate (generated by sonication [10 cycles of 10 s at 57% amplitude]) in the presence of 0.013 mg/mL brefeldin A for 16 hrs. PMA/ionomycin stimulation was included as positive control. At the end of the incubation, cells were surface stained with CD8b (Beckman Coulter), CD4, CD20, CD14, and HLA-DR (Biolegend); cells were then fixed and permeabilized followed by staining with IL-17, IFNγ (Biolegend), and TNF (Invitrogen). Data were acquired using an Attune NxT (Life Technologies, Carlsbad, CA) and analyzed using FlowJo software (TreeStar, Ashland, OR) with unstimulated background subtracted from stimulated samples.

### ELISA

IgG antibody titers against MAH were determined using enzyme-linked immunosorbent assay (ELISA). Plates were coated with 1 mg/mL MAH bacterial lysate overnight at 4°C. The plates were washed three times with 0.05% Tween-PBS. Heat-inactivated (56°C for 30 min) plasma samples were then added in 3-fold dilutions in duplicates for 1.5 h at room temperature. After washing with 0.05% Tween-PBS, Goat anti-monkey IgG (Fc) HRP (Brookwood Biomedical, Jemison, AL) was diluted 1:5000 then added to each well and incubated for 1–1.5 h. The plates were then washed three times with 0.05% Tween-PBS and incubated for 20 min in a solution containing o-phenylenediamine⋅2HCl (OPD) substrate (Sigma-Aldrich, St. Louis, MO) diluted in substrate buffer and 30% H_2_O_2_. The reaction was stopped by adding 1M HCl to each well. Absorbance at 490 nm was measured using the SpectraMax iD3 (Molecular Devices, San Jose, CA) plate reader. Each plate included a positive control which was used to normalize the data.

### Luminex

Levels of immune mediators in BALF supernatant were analyzed using the R&D 36-plex NHP XL Cytokine Premixed Kit (Bio-Techne, Minneapolis, MN). The following analytes were measured: BDNF, CCL2, CCL5, CCL11, CCL20, CD40L, CXCL2, CXCL10, CXCL11, CXCL13, FGF basic, G-CSF, GM-CSF, Granzyme B, IFNα, IFNβ, IFNγ, IL-1β, IL-10, IL-12 p70, IL-13, IL-15, IL-17A, IL-2, IL-21, IL-4, IL-5, IL-6, IL-7, IL-8, PDGF-AA, PDGF-BB, PD-L1, TGFα, TNF, VEGF. Samples were acquired using the MAGPIX xMAP (Luminex Corporation, Austin, TX). Data were analyzed using the Luminex Xponent software with an 8 point logistic regression curve.

### 16 s amplicon sequencing and bioinformatics analysis

Total DNA was extracted from nasal swabs using the DNeasy PowerSoil Pro Kit (Qiagen). Negative extraction controls were carried out with each extraction batch. Amplification of the hypervariable V4 region of the 16s rRNA gene was performed using the 515F/806R PCR primers; the forward primers were conjugated with a 12-bp barcode (Parada et al., 2016). Each reaction was run in duplicate and prepared with GoTaq master mix (Promega Corporation, Madison, WI) according to manufacturer’s instructions. Cycling conditions were: 94°C for 3 min, 37 cycles of 94°C for 45 s, 50°C for 1 min, and 72°C for 1 min, followed by a final cycle at 72°C for 10 min. Duplicate negative template PCR controls were also amplified. The PCR products were multiplexed using Quant-iT PicoGreen dsDNA Assay Kits and dsDNA Reagents (Thermo Fisher). The resulting library was then spiked with ∼15–20% PhiX and sequenced on an Illumina MiSeq. Raw FASTQ 16s rRNA gene amplicon sequences were processed using the QIIME2 analysis pipeline (Bolyen et al., 2019).

16S rRNA sequences were analyzed using the QIIME2 pipeline (Bolyen et al., 2019). Sequences were demultiplexed and biological samples yielded 220,338,098 raw reads (mean 228,804 ± 139,253 reads/sample; range 3,014,996 – 2). Sequences were denoised and filtered using DADA2 (trim-left-f 10, trim-left-r 10, trunc-len-f 280, trunc-len-r 190, n-threads 60) after which 82,863,456 reads were retained from biological samples (Callahan et al., 2016). MAFFT was used to align the sequence variants while FastTree2 was utilized to construct a phylogenetic tree (Katoh and Standley, 2013; Price et al., 2010). Taxonomy was assigned to sequence variants using q2-feature-classifier against the SILVA database (release 138) (Quast et al., 2013). Taxa identified as mitochondria, chloroplast, unassigned, and Mammalia were removed from subsequent analyses leaving 82,359,997 total reads from biological samples. The samples were rarified to 10,000 sequences per sample to normalize the sequencing depth between samples resulting in the exclusion of eight samples which fell below this threshold (*n* = 1 BAL-R D0, *n* = 1 BAL-R D183, *n* = 1 BAL-L D0, *n* = 2 Nasal D15, *n* = 1 Nasal D28, *n* = 1 Nasal D225, *n* = 1 Oral D7). QIIME2 was also used to generate alpha diversity metrics, which include richness (as observed ASV), Shannon evenness, and phylogenetic diversity, while beta diversity was estimated using weighted and unweighted UniFrac distances (Lozupone et al., 2011). The MaAsLin2 package was used to identify differentially abundant bacteria between groups with timepoint as the fixed effect and animal ID incorporated as a random effect (Mallick et al., 2021).

### Proteomics analysis

Macaque lung tissue was lysed (M-PER mammalian protein extraction reagent), reduced (5 mM dithiothreitol, 56 °C, 1 h), alkylated (10 mM iodoacetamide, 1 h, room temperature in the dark), and digested (Trypsin Gold, overnight) to generate peptides for mass spectrometry. Peptides were analyzed by nanoflow LC-MS/MS (Waters nanoAcquity UPLC coupled to a Thermo Orbitrap Fusion Lumos) using a reversed-phase gradient and data-dependent acquisition (DDA), with high-resolution MS1 scans and HCD-fragmented MS2 scans in the Orbitrap. Briefly, mobile phases were 0.1% formic acid in water (A) and in acetonitrile (B). Gradient (0.5 μL/min): 3–10% B (10 min), 10–30% B (10 min), 30–70% B (5 min), 70–95% B (3 min), 95→3% B (4 min), hold at 3% B (3 min); total run time 35 min. MS1 spectra acquisition: Orbitrap, resolution 120,000 (m/z 200), max IT 50 ms, AGC target 2E5. MS2: top 10 precursors, isolation width 1.6 Th, max IT 10 ms, AGC 2E4, HCD fragmentation (30% NCE), Orbitrap readout at 30,000 resolution. Dynamic exclusion: 30 s.

Raw DDA data were processed in FragPipe (v.22) for label-free quantification (LFQ), using MSFragger for database searching against the human proteome, followed by rescoring, protein grouping, and FDR filtering with standard FragPipe tools (MSBooster, Percolator, Philosopher, IonQuant). Differential protein expression between groups was assessed using FragPipe-Analyst: intensities were log2-transformed, missing values were imputed using the ‘Missing not At Random’ (MNAR) method, and pairwise comparisons were performed using empirical Bayes linear models via the R limma package. GO term analysis was performed with Metascape.

### Statistical analysis

Statistical analysis was performed using GraphPad Prism software (GraphPad Software Inc., La Jolla, CA). Normality was assessed using the Shapiro-Wilk test. Normal datasets were analyzed using a one- or two-way ANOVA with the Benjamini and Hochberg false discovery rate (FDR) correction method for multiple comparisons or a two-tailed, unpaired parametric Welch’s or Student’s *T*-test. Non-normal datasets were analyzed with mixed effects. We used a sphericity assay for the Luminex data.

## Results

### Antibiotic treatment and TNF blockade are associated with increased incidence of granulomas following MAH infection

We measured *M. avium* bacterial load in the right and left bronchoalveolar lavage fluid (BALF) of all animals in the antibiotic-treated, Inflectra-treated, and control groups using real-time quantitative PCR (qPCR) and by plating to solid growth medium. In line with previous studies (Cinco et al., 2024; Napier et al., 2025), both methods revealed peak bacterial burden 7 DPI that became undetectable at 44 DPI in both the left and right BALF (Figures 2A,B). qPCR showed a modest, but significantly larger peak bacterial load in antibiotic- and Inflectra-treated animals compared to control animals in the right BAL (Figure 2A). This was also observed by colony counts in antibiotic-treated animals (Figure 2B). Interestingly, although bacterial loads were detected 7 DPI in the left BAL following *M. avium* challenge of the right lung, no differences in bacterial load or colony numbers between all the groups were detected in the left BAL (Figure 2B).

**FIGURE 2:**
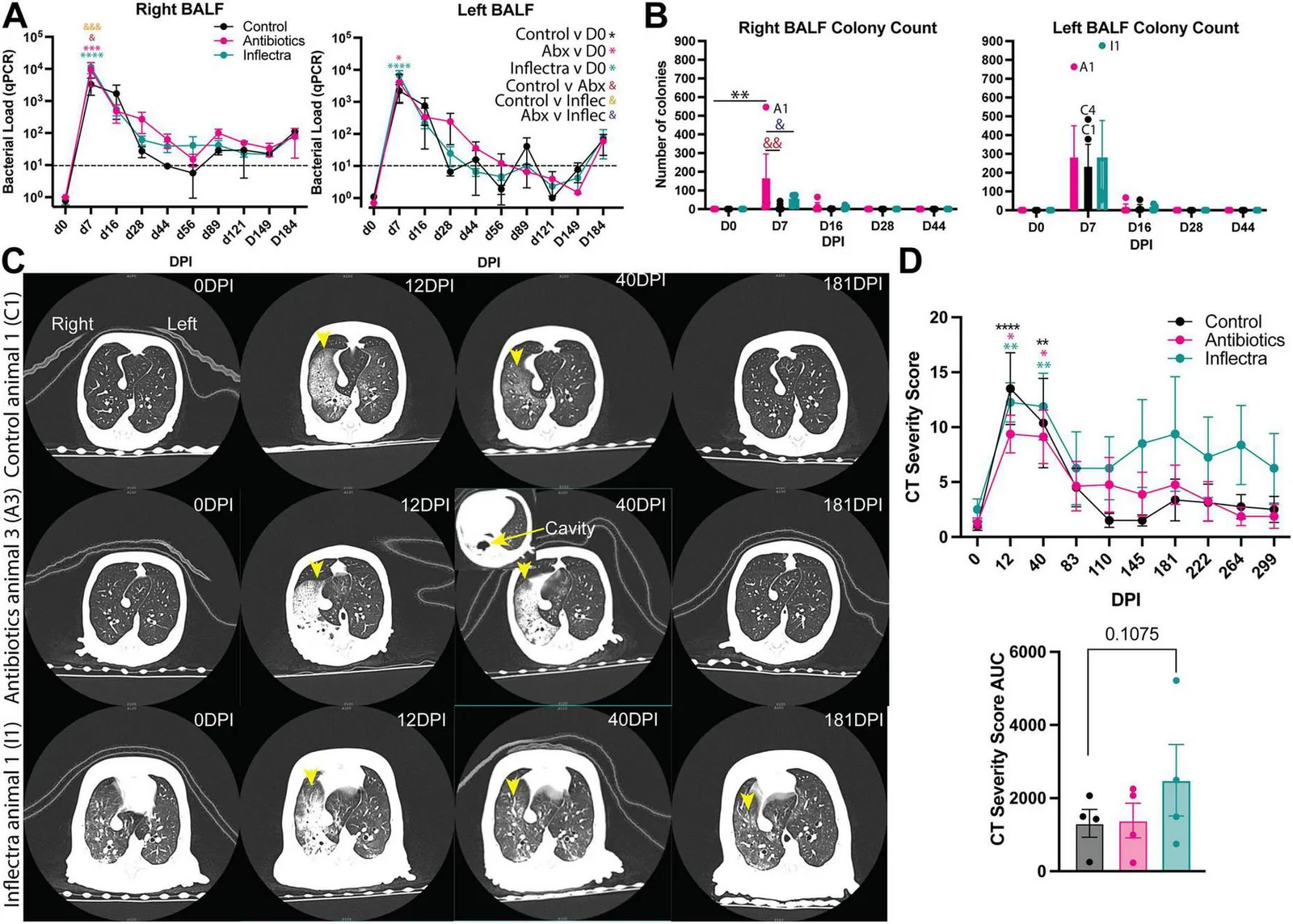
Increased bacterial load and heightened disease burden in some antibiotic- and Inflectra-treated animals. Bacterial load was measured in the BALF via **(A)** quantitative real-time PCR specific for the IS1311 sequence in *M. avium* and **(B)** plating on Lowenstein-Jensen agar plates to enumerate colony forming units (CFU) following 8 weeks of incubation. The dashed line represents the limit of detection. **(C)** Representative CT scans of antibiotic treated, control, and Inflectra treated animals before inoculation (0 DPI), at the height of inflammation (12 DPI) or of cavity formation (40 DPI), and during resolution (181 DPI). Yellow arrow heads point out inflammation. **(D)** CT scans were analyzed by a blinded cardiothoracic radiologist and reported as severity scores at the indicated days post inoculation (top) and as an area under the curve analysis (bottom). Data from the control group (C1–C4) are plotted in black, from the antibiotic group (A1–A4) are plotted in pink, and from the Inflectra-treated group (I1-I4) are plotted in teal. In panels A–C, data are average ± SEM. *p < 0.05, **p < 0.01, ***p < 0.001, ****p < 0.0001. ^&^*p* < 0.05, ^&&^*p* < 0.01, ^&⁣&&^*p* < 0.001, ^&⁣&⁣&&^*p* < 0.0001.

Computed tomography (CT) was performed throughout the infection and scored by a blinded cardiothoracic radiologist based on radiographic changes such as bronchiectasis, ground glass, and tree-in-bud opacities (Cinco et al., 2024). Consistent with the height of bacterial burden, CT severity scores peaked at 12 DPI in all 3 groups (Figures 2C,D and Supplementary Figures 1A–C). As observed in our prior studies, overt radiographic findings in control animals were resolved by 83 DPI and remained unremarkable for the remainder of the study (Figure 2C). One antibiotic-treated animal 3 (A3; female) developed a 16 * 20 mm cavity at 40 DPI, that expanded to 16 * 22 mm at 83 DPI and decreased to 4 * 5 mm by 299 DPI (Figure 2C). The remaining 3 antibiotic-treated animals showed comparable radiographic changes as the control group (Supplementary Figure 1B). One Inflectra-treated animal 1 (I1; male) showed persistent inflammatory changes, while the remaining 3 animals showed a comparable presentation as the controls (Figure 2C and Supplementary Figures 1C,D). Gross pathology findings showed areas of pulmonary consolidation in A3, A4, and I1 (Supplementary Figure 2).

Hemotoxylin and eosin (H&E) staining (Figures 3A–D) revealed that lungs of control animals were unremarkable at necropsy (Figure 3A and Supplementary Figure 3). Lung sections from antibiotic-treated animals showed inflammatory infiltrates in the right caudal lobe of antibiotic-treated animal A3, which had developed cavitary disease at day 40 (Figure 3B and Supplementary Figure 3). Finally, the right accessory and right middle lobes of animal I1, which showed persistent inflammatory changes by CT, showed granulomatous disease (Figure 3D). Quantification of immune infiltrates revealed a significant increase in the percentage of immune cells in the right compared to left lungs of Inflectra-treated animals driven by animal I1 (Figure 3E).

**FIGURE 3:**
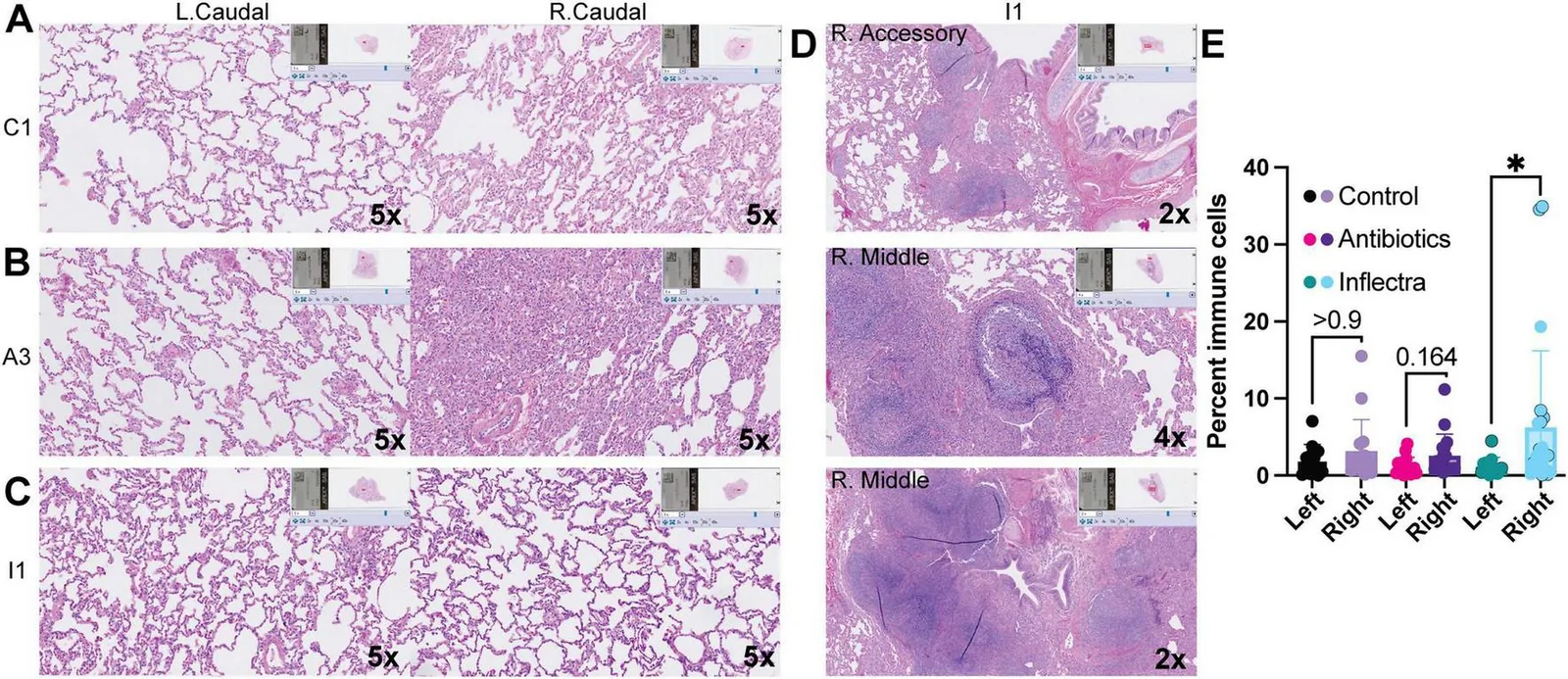
Antibiotic-treated animal A3 exhibits pulmonary consolidation and Inflectra-treated animal I1 exhibits granulomatous disease. H&E stains at the indicated magnifications of the left and right caudal lobes of panel **(A)** control animal 1, **(B)** antibiotic-treated animal 3, and **(C)** Inflectra-treated animal 1. **(D)** H&E stains at the indicated magnifications of the right accessory and middle lobes of animal I1. **(E)** Percent immune cells from the right and left lungs of antibiotic treated, control, and Inflectra-treated animals. Circles with black borders are from animal I1. *p < 0.05

### Antibiotic and inflectra treatment are associated with distinct inflammatory signatures in the lung following MAH infection.

We measured levels of immune mediators in the BALF by Luminex. To understand the impact of Inflectra and antibiotic treatment on the immune mediator landscape within the BALF, we performed a sPLSDA analysis at D0 (Figure 4A). Variate 1 primarily separated the Inflectra group from Control and Antibiotics groups while variable 2 separated Antibiotics and Inflectra groups from the control group. Levels of IL-6, CCL5, CXCL10, CCL20, CCL2, IL-4, IFNα, IL-17, IL-2 and IL-6 delineated the control group at D0 while IL-21, PD-L1, CD40L, FGF basic, and VEGF delineated the Antibiotics group and CCL11 separated the Inflectra group. These findings suggest that the response in the control group is characterized by mediators associated with both innate and adaptive immunity whereas Antibiotics group appears slanted toward tissue repair after the treatment.

**FIGURE 4:**
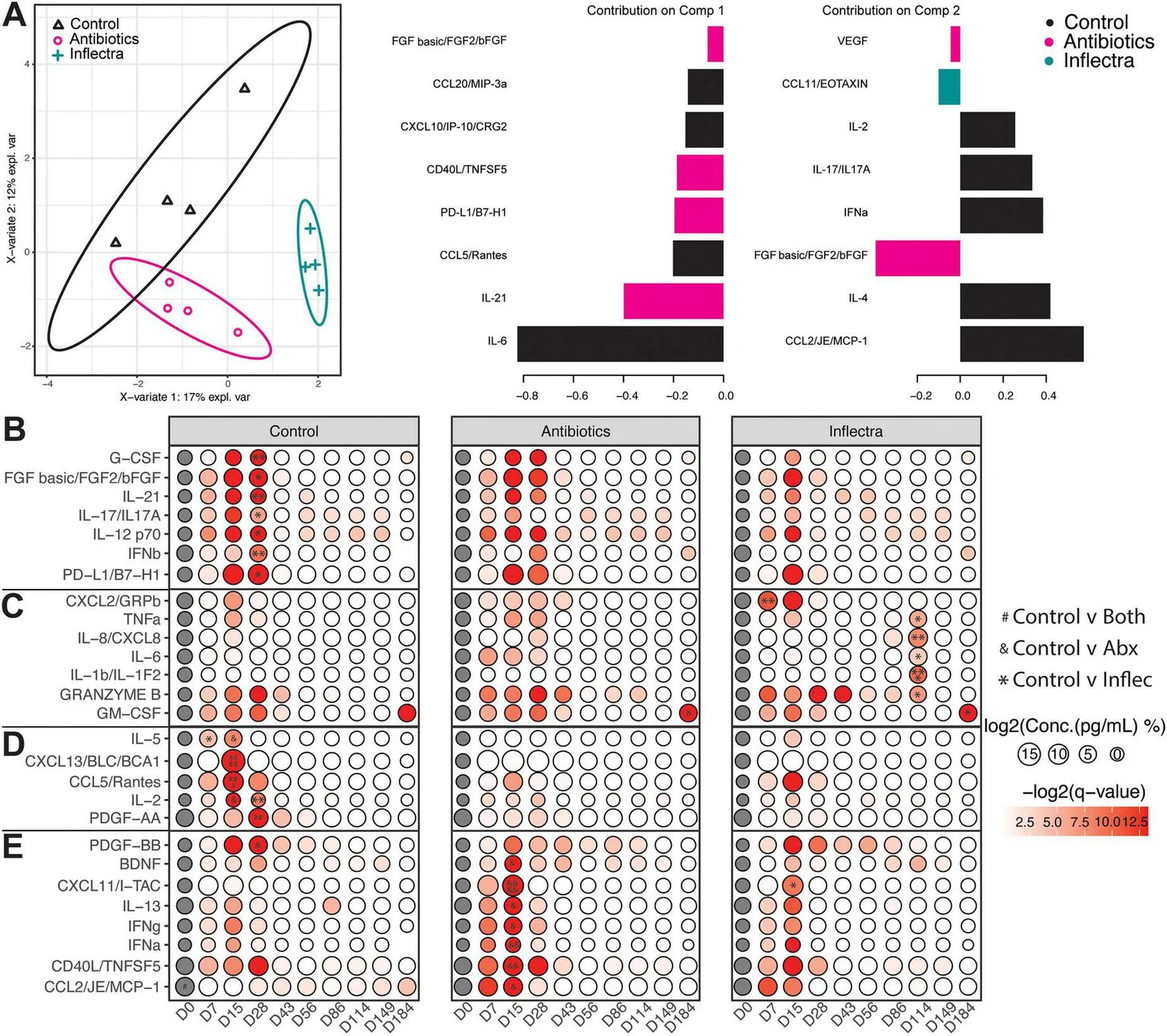
Overexpression of mediators characteristic of a Th1 response in antibiotic-treated animals and of mediators characteristic of an acute-phase response in Inflectra-treated animals. **(A)** sPLS-DA plot of immune mediators in D0 BALF supernatants from right lungs determined by Luminex in control, antibiotic-treated, and Inflectra-treated groups. Axes show the indicated components and explained variance. Bar plot represent significant mediators driving responses in each group. **(B–E)** Bubble plot of the differentially abundant immune mediators in BALF supernatants determined by Luminex assays at the indicated timepoints in the right lungs of the control, antibiotic-treated, and Inflectra-treated groups. The size of the bubble represents the concentration of the immune mediator while color represents the significance of the change. &indicates a significant difference between the control and antibiotic groups. *indicates a significant difference between the control and Inflectra groups *p < 0.05, **p < 0.01, ***p < 0.001. ^&^*p* < 0.05, ^&&^*p* < 0.01, ^&⁣&⁣&&^*p* < 0.0001, ^##^*p* < 0.01, ^###^*p* < 0.001, ^####^*p* < 0.0001.

We next longitudinally measured levels of immune mediators in the BALF by Luminex (Figures 4B–E). Growth factors G-CSF and FGF basic as well as pro-inflammatory mediators IL-21, IL-17, IL-12, IFNβ, and anti-inflammatory PD-L1 were higher in the control group 28 DPI compared to the Inflectra-treated group (Figure 4B). Levels of chemoattractants IL-5 (eosinophil), CXCL13 (B cell), CCL5 (T cells and monocytes) as well as IL-2 (T cell modulator) and PDGF-AA (cell division) were increased in controls compared to both Inflectra- and antibiotic-treated animals 7–28 DPI (Figure 4D). Finally, PDGF-BB (vascular remolding and cell proliferation) levels were increased in the control versus the antibiotic-treated group at 28 DPI.

On the other hand, the Inflectra-treated group had higher levels of CXCL2 (leukocyte chemoattractant) at 7 DPI (Figure 4C), as well as higher levels of TNF (Th1 response), IL-8 (neutrophil chemoattractant), IL-6 (acute phase response), IL-1β (induces IL-6 and TNF), and Granzyme B (cytotoxic T lymphocyte protease) at 114 DPI compared to control animals (Figure 4B). The granulocyte growth factor GM-CSF level was increased in BALF of both Inflectra- and antibiotic-treated animals 184 DPI (Figure 4C). The antibiotic-treated group exhibited higher levels of BDNF (growth factor), CXCL11 (T cell and NK cell chemoattraction), IL-13 (mucus secretion), IFNγ, IFNα, CD40L (co-stimulatory factor), and CCL2 (monocyte/macrophage chemoattractant) 15 DPI compared to controls (Figure 4E). Thus, overexpression of mediators characteristic of the innate immune response was detected in the Inflectra-treated group (increased IL-8 and IL-6) while the antibiotic-treated group exhibited overexpression of molecules involved in adaptive immune (CXCL11, CD40L) and Th1 (increased IFNγ) responses.

### Antibiotic or inflectra treatment are associated with distinct immune cell composition in the lung following MAH infection.

Macrophage and T cell frequencies and MAH-specific responses were longitudinally evaluated by flow cytometry using our previously published method (Cinco et al., 2024). Right and left BALF in all animals was composed of ∼60% alveolar macrophages (AMs) at the time of infection (Figure 5A and Supplementary Figure 4A). AM relative abundance decreased dramatically at 7 DPI in the right lungs (site of inoculation) of antibiotic- and Inflectra-treated animals, and 28DPI in controls, returning to pre-infection levels after 149 DPI in controls and Inflectra-treated animals but not antibiotic-treated animals. Loss of AM in the left BAL was only evident in the antibiotic- and Inflectra-treated groups returning to pre-infection levels after 86 DPI (Supplementary Figure 4A).

**FIGURE 5:**
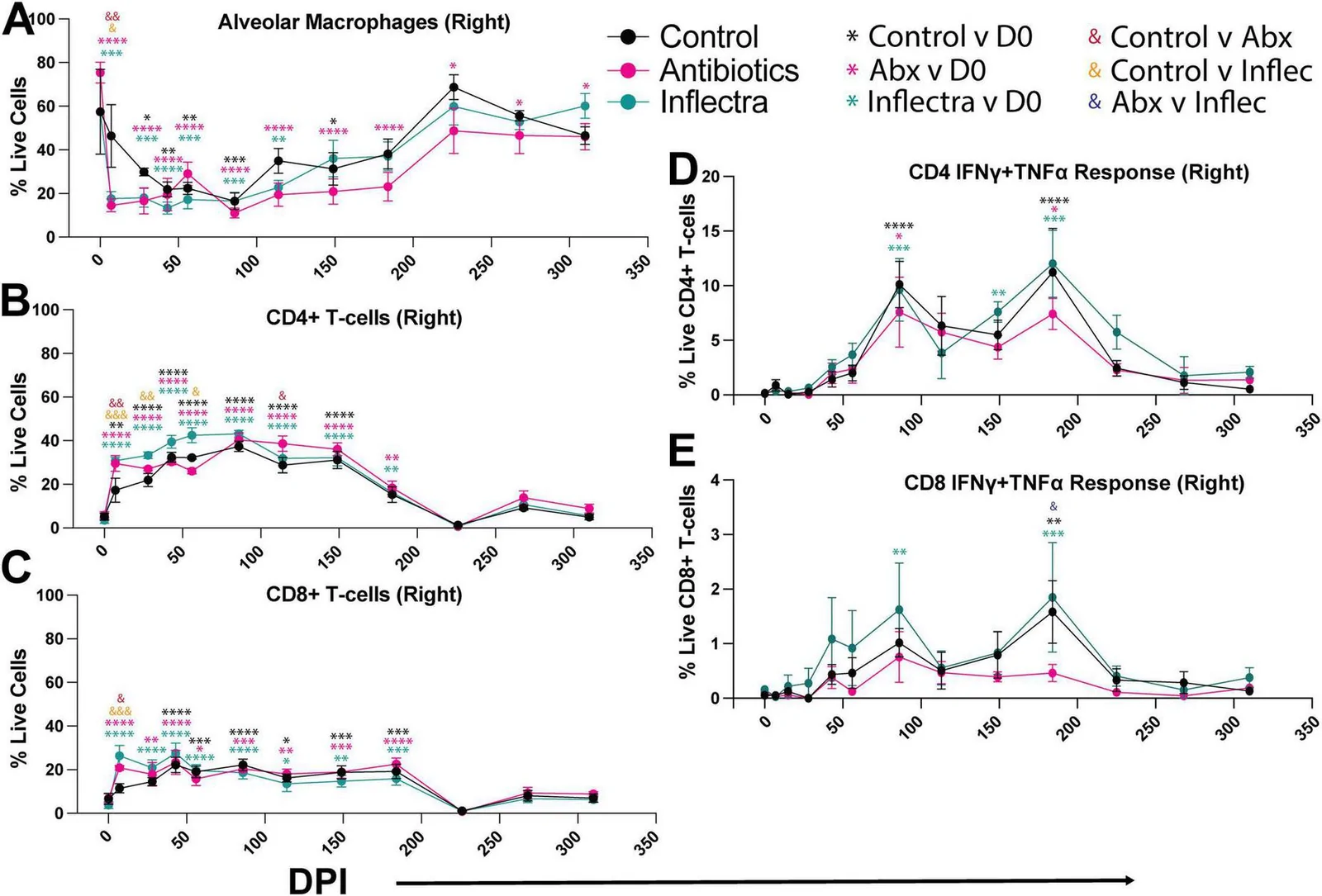
Inflectra-treated animals have modestly more T cell influx. BALF samples were collected longitudinally from the right lungs at the indicated DPI and flow cytometry was performed to enumerate the relative frequency within live cells of panel **(A)** alveolar macrophages; **(B)** CD4+ T-cells, **(C)** CD8+ T cells, **(D)** MAH-specific IFNγ+ TNF+ CD4 T-cells, and **(E)** MAH-specific IFNγ+ TNF+ CD8 T-cells. The MAH-specific CD4 and CD8 T-cell response was determined by intracellular cytokine staining following stimulation with 1 mg/mL MAH lysate. Data are average ± SEM. Two-way ANOVA statistical tests were performed to evaluate within group changes compared to 0 DPI (denoted by asterisks) and between group changes at each timepoint (denoted by ampersands). *p < 0.05, **p < 0.01, ***p < 0.001, ****p < 0.0001.

The loss of AMs starting at 7 DPI was concurrent with an influx of CD4+ and CD8+ T cells (Figures 5B,C and Supplementary Figure 4B,C). The antibiotic- and Inflectra-treated groups showed an earlier CD4 and CD8 T cell influx in the right lung than controls (7 DPI), but T cell levels in all groups were elevated between 28 DPI and 226 DPI (Figures 5B,C). We then investigated frequency of antigen-specific T cells using intracellular cytokine staining following stimulation with MAH lysate. Interestingly, MAH-specific CD4 T cell responses were only evident starting 86 through 184 DPI reaching statistical significance only sporadically (Figure 5D). MAH-specific CD8 T cells were detected in the right lung of Inflectra-treated animals 86 and 184 DPI and in control animals at 184 DPI but not in the antibiotic-treated group (Figure 5E).

Within the left lungs, CD4 T cell levels were elevated in the Inflectra-treated group compared to controls 28 and 86 DPI (Supplementary Figure 4B). CD8 T cells were higher in the antibiotic-treated group 28–43 DPI and in the Inflectra-treated group 28–86 DPI but were not significantly increased in the control animals (Supplementary Figure 4C). CD4 T cell responses in the left lung were detected with similar kinetics as the right lung except for the control animals at 86 DPI likely due to the large standard deviation within the control group (Supplementary Figure 4D). The Inflectra-treated group exhibited more CD4 and CD8 T cells in the left lung 184 DPI compared to control and antibiotic groups (Supplementary Figures 4D,E). MAH antigen-responsive CD8 T cells were only detected in left lungs of Inflectra-treated animals 86 and 184 DPI (Supplementary Figure 4E).

We measured MAH-specific IgG antibody titers by ELISA in plasma and BALF supernatants. Circulating MAH-specific IgG antibodies were slightly higher in plasma from control animals compared to the antibiotic-and Inflectra-treated groups (Supplementary Figure 4F). No significant differences in IgG titers were observed in the right BALF (Supplementary Figure 4G). Collectively, these data suggest that antibiotic- and Inflectra-treated animals have more T cell influx during their immune response to MAH.

### Lung microbiome compositional shifts with antibiotic and inflectra treatment as well as infection

To profile the BALF, oral, nasal and fecal microbiomes, we performed 16S rRNA sequencing. Weighted and unweighted principal coordinate analysis (PCoA) of control animals revealed distinct separation with each sample type falling into unique quadrants (Figure 6A and Supplementary Figure 5A). The Inflectra-treated samples largely resembled controls, but the BALF samples from antibiotic-treated animals were distinct (Figure 6A). We investigated the top 10 most abundant genera in the right MAH-infected BALF of each experimental group (Figure 6B). As we previously observed (Cinco et al., 2024; Napier et al., 2025), *Tropheryma* dominated the lung microbiome of the control and Inflectra-treated groups before infection and was reduced following infection (Figure 6B and Supplementary Figure 5B). Animals in the antibiotic-treated group that received a dose of amikacin and vancomycin 3 days before infection had reduced *Tropheryma* 0 DPI compared to the control and Inflectra groups (Figure 6B). Interestingly, antibiotic-treated animal A3 had a relative abundance of *Tropheryma* of 0.39% at baseline (before antibiotic treatment and MAH infection). A4, which showed signs of lung consolidation, had a *Tropheryma* relative abundance of 6.55%, while A1 and A2 showed minimal radiographic changes had relative abundances of 99.29% and 56.58%, respectively. These observations are in line with our prior reports that suggested that relative abundance of *Tropheryma* negatively correlates with disease severity (Cinco et al., 2024; Napier et al., 2025). The overall microbial load in the right lung of control and antibiotic-treated animals did not drastically change due to antibiotic treatment or infection (Supplementary Figure 5C). In contrast, the microbial load of Inflectra-treated animals was significantly increased by 85 and 113 DPI (Supplementary Figure 5C). The antibiotic-treated group exhibited significant changes in the overall diversity of their right lung microbiome 86 DPI and in the left lung 15, 28, 86, and 149 DPI (Supplementary Figure 5D). In addition and in line with the qPCR and culture data, reads mapping to *Mycobacteria* species were more abundant at 7 DPI in the antibiotic-treated animals compared to the Inflectra-treated and control animals (Figure 6C).

**FIGURE 6:**
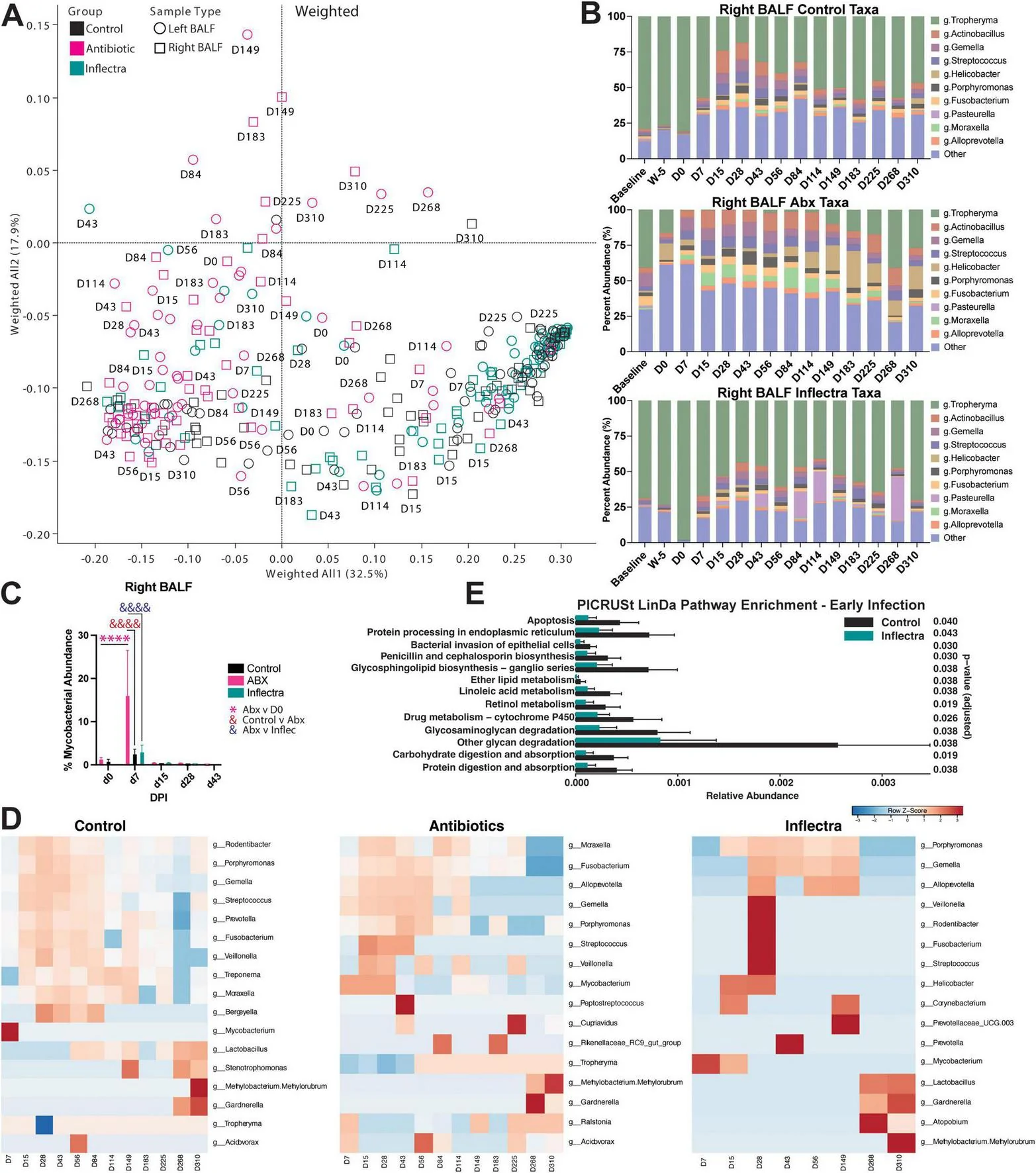
Lung *Tropheryma* abundance is decreased by antibiotic treatment but increased by Inflectra treatment. **(A)** The weighted principal coordinate analysis (PCoA) plot of the left and right BALF at the indicated DPI. **(B)** Bar plots displaying the dominant phyla in the right BALF and their percent relative abundances at the indicated timepoints. Baseline is the timepoint before any treatments were ever done. **(C)** Percent *Mycobacterium* abundance as measured by 16S rRNA sequencing in the right BAL. **(D)** Heatmaps derived from MaAsLin analysis of the right BALF microbiome profiling. The color represents the difference in relative abundance compared to D0 timepoint. **(E)** Phylogenetic investigation of communities by reconstruction of unobserved states (PICRUSt) using linear models for differential abundance analysis (LinDA) at early-infection timepoints (7–86 DPI) between control and Inflectra-treated animals. Pathway enrichments were filtered based on a log2 fold change > 1.5 and adjusted *p*-value < 0.05. ****p < 0.0001, ^&⁣&⁣&&^*p* < 0.0001.

A Microbiome Multivariable Associations with Linear Models (MaAsLin2) analysis was performed on the right BALF to investigate the longitudinal changes of specific microbes following MAH inoculation compared to D0 within each group (Figure 6D). In the control group, reads mapping to *Mycobacterium* genera were detected at D7. Additionally, infection triggered a self-limited decompartmentalization characterized by a broad, gradually building bloom of oral/upper respiratory tract (URT) commensals (*Veillonella, Fusobacterium, Porphyromonas, Streptococcus*) peaking D15–D56 and largely resolving thereafter, with only a late-phase emergence of additional taxa (*Lactobacillus, Stenotrophomonas, Methylobacterium, Gardnerella*, D268–310). Notably, *Tropheryma* shows only a single, isolated, sharp decrease at D28. In the Antibiotics group, reads mapping to *Mycobacterium* were detected for longer duration (D7 - D28) than the control group, consistent with delayed bacterial clearance in this group. The same URT bloom observed in the control group was also broader and of higher-amplitude. Isolated sharp single-timepoint spikes appear for *Peptostreptococcus* (D43) and *Cupriavidus* (D225), and the gut-associated Rikenellaceae recurs at both D84 and D183, a signal not seen in the control group. Moreover, *Tropheryma* abundance was decreased for a longer duration compared to the control group (D7 to D43). In the Inflectra group, reads mapping to *Mycobacterium* were also detected for longer duration (D7 - D15) than the control group. The oral/URT bloom was delayed relative to the control group but was sharper with a single synchronized spike at D28 (*Veillonella, Fusobacterium, Streptococcus, Rodentibacter* all peaking together) rather than a gradual rise over several weeks. We note a gut-associated genus (*Helicobacter*) appearing at D15, earlier than any oral/URT taxon in this group and not observed in the other groups, a possible signature of impaired gut barrier integrity under TNF blockade.

We subjected the BALF 16S rRNA amplicon sequencing data to phylogenetic investigation of communities by reconstruction of unobserved states (PICRUSt) to infer the functional capabilities of the lung microbiome (Langille et al., 2013; Figure 6E). We performed comparisons at pre-infection, early-infection (7–84 DPI), and late-infection (114–310 DPI) between control and antibiotic-treated groups as well as between control and Inflectra-treated groups. Only control versus Inflectra-treated groups at early-infection showed significant differences (log2 fold change > 1.5 and adjusted *p*-value < 0.05) when using linear models for differential abundance analysis (LinDA). Specifically, enrichment to pathways associated with protein processing, bacterial invasion of epithelial cells, biosynthesis of penicillin, cephalosporin, and glycosphingolipid as well as metabolism of ether lipid, linoleic acid, and retinol was predicted to be decreased in the Inflectra-treated group (Figure 6E). These data suggest that Inflectra treatment coupled with MAH infection may suppress several biosynthesis, metabolism, and absorption pathways.

The nasal microbiome of antibiotic-treated animals was initially more diverse than the other groups at 7 DPI (Supplementary Figure 6A). *Psychrobacter* in the nasal community was most affected by antibiotic administration but recovered to baseline levels by 7 DPI (Supplementary Figure 6A). The oral microbiome of the antibiotic group exhibited more diversity 28, 43, 114, and 184–268 DPI (Supplementary Figure 6B). The fecal microbiome diversity of the antibiotic group was lower than that of the Inflectra-treated group 0 DPI (Supplementary Figure 6C). Fecal diversity was increased in all groups 15–56 and 226 DPI and returned to baseline by 310 DPI (Supplementary Figure 6C).

### Lesion-adjacent tissue from antibiotic- and inflectra-treated animals display proteome changes indicative of inflammatory changes

We performed proteomic data acquisition and analysis on healthy lung tissue from the left lower lobe of control C2, the right upper lobe of Inflectra-treated I1 that was adjacent to a lesion, the right middle lobe of Inflectra-treated I2 near a site of inflammation on CT, the right upper lobe of antibiotic-treated A3 that was adjacent to a lesion, and the right middle lobe of A3 (Figures 7A,B and Supplementary Figures 7A,B). Comparison of lung tissue from C2 to the right upper lung of I1 that was adjacent to a lesion showed increased expression of 43 proteins in C2 relative to I1 and 50 proteins overexpressed in I1 compared to animal C2 (Figure 7A). Proteins more abundant in C2 were suggestive of an antibacterial humoral response (IGLV3-9, IGLV1-47, JCHAIN, IGHM) as well as lung tissue remodeling (ALDOC, POSTN, NID1, ZYX). On the other hand, the proteome of I1 was enriched in proteins associated with antigen presentation (HLA-E, HLA-DRB4, HLA-DRA), tissue remodeling (KRT18, COL14A1, TGFBI, PLVAP, and LAMA3), protein depolymerization (PSMB10), reactive oxygen species (SOD3), and hypoxia (HYOU1) (Figure 7A). Similarly, the sample from I2 was enriched in proteins related to inflammation and antigen presentation (IGSF8, IGHG1, HLA-DRA, HLA-DRB4, TNFAIP2, IGLV3-16, S100A8, GBP4, S100A9, MRC1, S100A14) and wound response (EPS8, CLDN5, CADM1, PDGFRB, ITGAV, CD9, MRC1) (Supplementary Figure 7A).

**FIGURE 7:**
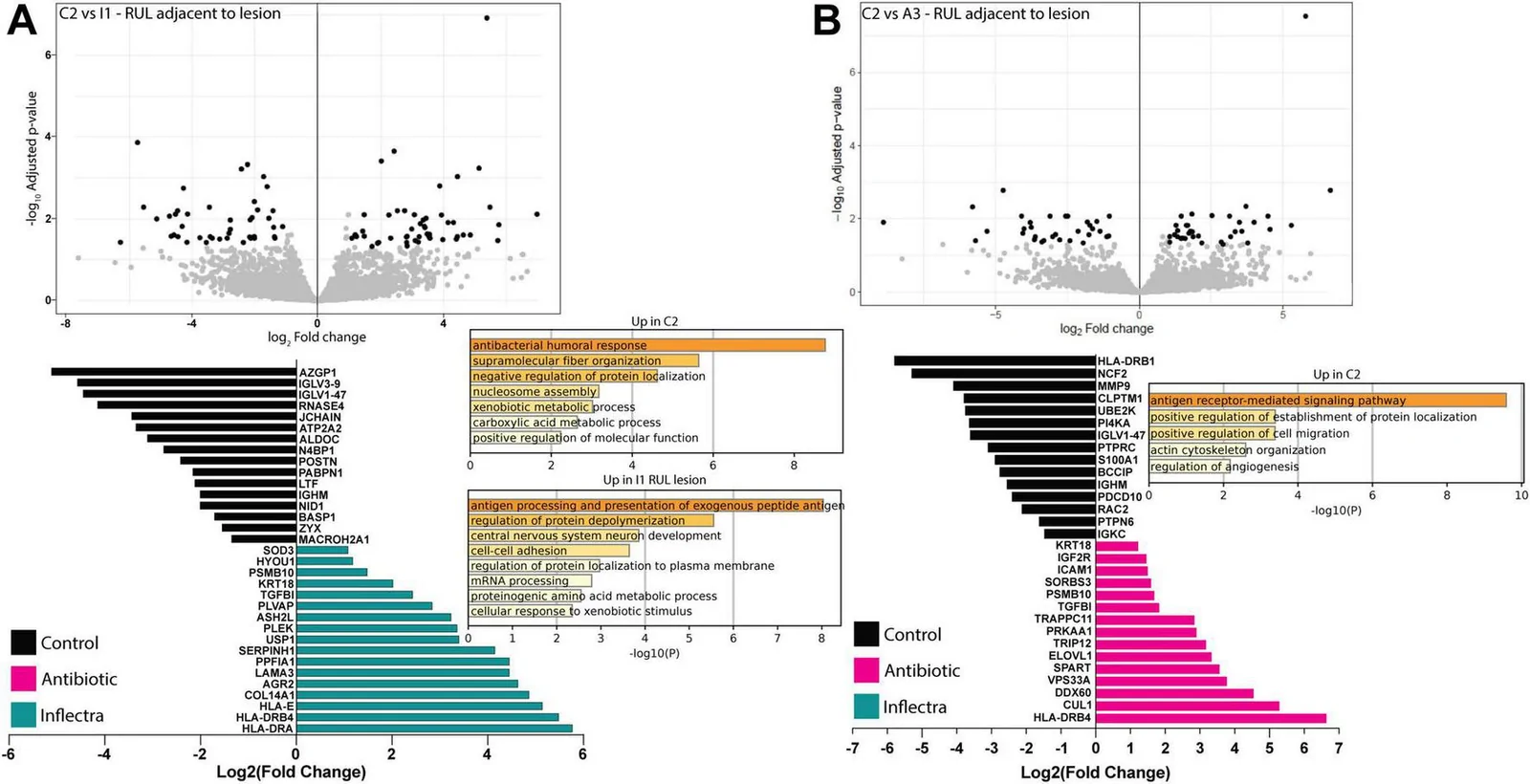
Proteome changes in antibiotic- and Inflectra-treated animals indicative of lack of humoral immune responses compared to control animals. Differentially abundant proteins and Gene Ontology (GO) enrichment bar plots in the left lower lung tissue of panel **(A)** animal C2 compared to the right upper lobe that was near a lesion of animal I1 and **(B)** animal C2 compared to the right upper lobe that was near a lesion in A3.

Comparison of lung tissue from animal C2 to the lesion in animal A3 revealed 32 overexpressed proteins in C2 and 35 overexpressed in A3 (Figure 7B). The control lung was enriched in proteins involved in antigen-receptor mediated signaling (HLA-DRB1, UBE2K, PI4KA, PTPRC, PTPN6) and the humoral response (IGLV1-47, IGHM, IGKC). Abundant proteins in A3 were involved in tissue organization and development (KRT18, IGF2R, TGFBI, SPART, CUL1), cellular adhesion (ICAM1, SORBS3), autophagy (PRKAA1, VPS33A), and immune responses (PSMB10, DDX60, HLA-DRB4) (Figure 7B). The tissue from the right middle lobe of animal A3 showed higher abundances of proteins important for antigen presentation (HLA-DRB4, HLA-DRA) and tissue repair (SERPINH1, ITGA6, IGF2R, ITGAV, TGFBI, PDGFRB) (Supplementary Figure 7B).

## Discussion

Clinical and genome-wide association studies (GWAS) indicate that effective immunity against NTM requires robust Th1 T cell responses (McGarvey and Bermudez, 2002). Mutations in key Th1 pathway genes such as IL-12 subunits, STAT1, and interferon stimulated genes are linked to increased susceptibility to NTMPD in children (Chen et al., 2017; Haverkamp et al., 2014; Pierre-Audigier et al., 1997; Wu and Holland, 2015). Therapies that block TNFα further elevate this risk (Winthrop et al., 2013; Winthrop et al., 2008). Consistent with this, peripheral blood mononuclear cells from individuals with NTMPD produce less IFNγ and TNFα than those from healthy controls (Gold et al., 2010; Winthrop et al., 2008). Additionally, a dysbiotic respiratory microbiome has been implicated as a host factor in NTMPD, as reflected by distinct community profiles in healthy versus NTM-infected lungs (Dickson et al., 2017; Philley et al., 2019; Sulaiman et al., 2018). Indeed, in a cohort of women with a history of breast cancer, the sputum from women with NTMPD compared to those without show reduced abundance of *Leptotrichia*, *Streptococcus*, and *Veillonella* (Philley et al., 2019). In addition, BALF from patients with NTMPD is enriched in *Ralstonia*, *Clavibacter*, and *Enterobacter* relative to individuals with non-CF bronchiectasis (Sulaiman et al., 2018). Collectively, this work suggests that Th1 immune responses and the lung microbiome are important factors during NTMPD. Therefore, we examined the role of the TNF immune signaling and the lung microbiome composition on pulmonary MAH infection in rhesus macaques using Inflectra and antibiotic treatment respectively. Inflectra treatment was associated with development of pulmonary granulomatous disease in one of four animals, while inhaled antibiotic treatment was associated with cavitary disease in one of four animals. None of the four control animals developed granulomatous or cavitary disease. These observations suggest that TNF responses and a healthy microbiome are important for protection against NTMPD.

In all groups, bacterial load was highest at 7 DPI followed by a rapid clearance of MAH. Animals treated with antibiotics or Inflectra showed higher level of MAH at 7 DPI as quantified through either culture or qPCR. These results suggest either greater bacterial colonization or decreased ability of the host to control early infection resulting in higher MAH. Disease outcomes for animals I1 (severe inflammation and granulomatous disease) and A3/A4 (cavitary disease, consolidation), suggest that the host immune response is impaired in animals treated with Inflectra or inhaled antibiotics.

Several important differences in immune mediator production between the control and treated cohorts were noted. Early in infection, control animals had significantly higher levels of pro-repair growth factors (G-CSF) (Chandra et al., 2026; Prudovsky, 2021), anti-inflammatory PD-L1 (Chen et al., 2023), as well as crucial cytokines for initiating type 1 immune programs (IL-12) (Sun et al., 2015) and lung immunity (IL-17) (Ritzmann et al., 2022). On the other hand, the Inflectra-treated animals had a brief spike of TNF production in the right lungs compared to the control animals at 114 DPI, a potential indicator of a compensatory mechanism to the Inflectra treatment received at 105 DPI (Berkhout et al., 2019). Inflectra-treated animals also overexpressed IL-6, IL-8, IL-1β at the 114 DPI timepoint, suggesting that these animals may have relied on a non-Th1 immune response to MAH (Zeng et al., 2018). Proteomic analysis indicated upregulation of proteins involved in antigen processing and presentation pathways in Inflectra-treated I1, in contrast to the humoral response signatures seen in control C2. While these findings are exploratory, lesion-level observations from single representative animals rather than evidence of a group-wide humoral immune deficit, they suggest that in this Inflectra-treated animal, the innate immune response is stimulated but not the adaptive immune response (Bruns et al., 2009; Fremond et al., 2005).

Antibiotic-treated animals showed increased levels of CXCL11, IFNα, IFNγ and CD40L compared to control animals at 15 DPI, suggesting a stronger type 1 immune response (Zeng et al., 2018). Despite the increased cytokines in the BALF of antibiotic-treated animals at 15 DPI, upregulation of HLA-DRB4, B2M (antigen presentation) was the only proteomic evidence of immune involvement in the lung tissue of animal A3 at necropsy (310 DPI). Instead, several tissue repair and remodeling proteins such as IGF2R, TGFBI, and PDGFRB that could contribute to the cavitary resolution were increased (Desai and Cardoso, 2002).

Previous findings indicated a possible important role for the respiratory microbiome in the development of NTMPD. We previously observed a negative correlation between relative abundance of *Tropheryma* and severity of radiographic changes post MAH infection in this model (Cinco et al., 2024; Napier et al., 2025). Antibiotic-treated animals had a significantly lower *Tropheryma* abundance at the onset of infection that was further reduced after MAH infection and did not rebound to the same extent as seen in the control animals. Notably, antibiotic-treated animal A3 that developed cavitary disease had the lowest relative abundance of *Tropheryma* at baseline (before antibiotic treatment and MAH infection) compared to the other antibiotic-treated animals. In control animals, the initial *Tropheryma* abundance, depletion after MAH infection, and recovery by the end of the study was consistent with our previous work (Napier et al., 2025).

While the data presented here advances our understanding of host-microbe interactions during NTM infection, the study is not without limitations. The low sample size of four macaques in each group, and the observed pathology was limited to a single animal in each treatment group, highlighting the exploratory, case study nature of these findings. Additionally, we only impaired young animals’ ability to control MAH infection by inhibiting TNF signaling or the lung microbiome alone. We did not directly measure the extent, duration, or cellular and compartment specificity of functional TNF neutralization achieved by Inflectra treatment, including the relative blockade of membrane-bound versus soluble TNF, or which specific cell types were most affected. These pharmacodynamic parameters represent an important target for future work. Single-cell transcriptomic or high-parameter cytometric profiling of longitudinal BALF and terminal lesion tissue would help resolve which specific innate and adaptive immune subsets drive the divergent phenotypes observed across treatment groups. Finally, this study relied on 16S rRNA amplicon sequencing as a result, our conclusions about microbial functional capacity remain inferential. Shotgun metagenomics, meta transcriptomics, and/or metabolomics would allow direct confirmation of the functional predictions we inferred from taxonomic data alone, strengthening the link between compositional microbiome shifts and their functional consequences for host-pathogen interactions. Finally, our observations indicate that impaired TNF signaling or shifts in lung microbiome alone are not sufficient to recapitulate age-mediated increased susceptibility to NTM infection. It is possible that combining the two treatments in future studies would better replicate the disease outcome observed in aged macaques. These findings further support the notion that increased susceptibility to NTMPD is multifactorial and that additional changes in epithelial cells and mucociliary escalator are likely critical components.

## Data Availability

The datasets presented in this study can be found in online repositories. The names of the repository/repositories and accession number(s) can be found below: https://www.ncbi.nlm.nih.gov/, PRJNA1398294.

## Supplementary material

Supplementary Figure 1 Representative computed tomography scans. CT scans of the **(A)** control (C2, C3, and C4), **(B)** antibiotic (A1, A2, and A4), and **(C)** Inflectra (I2, I3, and I4) groups at 0, 13, 40, and 180 DPI. The yellow arrowheads note inflammation. **(D)** Individual CT severity scores of I1, I2, I3, and I4 (top) and A1, A2, A3, and A4 (bottom).

Supplementary Figure 2 Gross pathology. Gross pathology of lungs from **(A)** C1–C4, **(B)** A1–A4, and **(C)** I1–I4. White boxes indicate areas of pulmonary consolidation.

Supplementary Figure 3 Supplemental H&E stains. Representative H&E stains at the indicated magnification that were acquired for the left caudal and right caudal lobes of animals C2–C4, A1, A2, A4, I2–I4.

Supplementary Figure 4 Immune cell influx in left lung. BALF samples were collected from the left lungs at the indicated DPI and flow cytometry data were acquired. The relative frequency within live cells of panel **(A)** alveolar macrophages; **(B)** CD4+ T-cells, **(C)** CD8+ T cells, **(D)** MAH-specific IFNγ+ TNF+ CD4 T-cells, and **(E)** MAH-specific IFNγ+ TNF+ CD8 T-cells. The MAH-specific CD4 and CD8 T-cell response was determined by intracellular cytokine staining following stimulation with 1 mg/mL MAH lysate. The MAH-specific IgG end-point titer averages in the **(F)** plasma and **(G)** right BALF. The dashed lines represent the limit of detection. In all panels, the average and standard error of the mean for all animals in the group is shown. A 2-way ANOVA analysis was performed to compare timepoints within the experimental groups to their respective 0 DPI baseline (black, pink, or teal stars), or to compare between groups at each timepoint (red, orange, and blue ampersands). **p* < 0.05, ^**^*p* < 0.01, ^***^*p* < 0.001, ^****^*p* < 0.0001.

Supplementary Figure 5 Microbiome profiling of the BALF. **(A)** The weighted principal coordinate analysis (PCoA) plot of the left and right BALF, fecal, nasal, and oral swabs at the indicated DPI. **(B)** Bar plots of the dominant phyla in the left BALF of each experimental group. **(C)** Total bacterial load was measured in the right BALF via quantitative real-time PCR specific for the 16S rRNA gene sequence. **(D)** Amplicon sequence variants (ASVs) in the right and left BALF. Two-way ANOVA statistical tests were performed to evaluate within group changes compared to 0 DPI (denoted by asterisks) and between group changes at each timepoint (denoted by ampersands). **p* < 0.05, ^**^*p* < 0.01, ^***^*p* < 0.001, ^****^*p* < 0.0001.

Supplementary Figure 6 Microbiome profiling of the nasal, oral, and fecal swabs. Amplicon sequence variants (ASVs) and dominant phyla in the **(A)** nasal, **(B)** oral, **(C)** and fecal swabs.

Supplementary Figure 7 Additional lung proteomic comparisons. Differentially abundant proteins and Gene Ontology (GO) enrichment bar plots in the left lower lung tissue of panel **(A)** animal C2 compared to the right middle lobe that was near an infection site of animal I1 and **(C)** animal C2 compared to the right middle lobe that was clear in A3.

## References

[B1] Anand K. Burns E. A. Ensor J. Rice L. Pingali S. R. (2020). mycobacterial infections with ruxolitinib: A Retrospective pharmacovigilance review. *Clin. Lymphoma. Myeloma Leuk*. 20, 18–23. 10.1016/j.clml.2019.08.008 31699655

[B2] Andréjak C. Nielsen R. Thomsen V. Ø Duhaut P. Sørensen H. T. Thomsen R. W. (2013). Chronic respiratory disease, inhaled corticosteroids and risk of non-tuberculous mycobacteriosis. *Thorax* 68, 256–262. 10.1136/thoraxjnl-2012-201772 22781123

[B3] Berkhout L. C. l’Ami M. J. Ruwaard J. Hart M. H. Heer P. O. Bloem K. et al. (2019). Dynamics of circulating TNF during adalimumab treatment using a drug-tolerant TNF assay. *Sci. Transl. Med.* 11:eaat3356. 10.1126/scitranslmed.aat3356 30700574

[B4] Bolyen E. Rideout J. R. Dillon M. R. Bokulich N. A. Abnet C. C. Al-Ghalith G. A. et al. (2019). Reproducible, interactive, scalable and extensible microbiome data science using QIIME 2. *Nat. Biotechnol.* 37:1091. 10.1038/s41587-019-0209-9 31341288 PMC7015180

[B5] Boukhvalova M. S. Yim K. C. Kuhn K. H. Hemming J. P. Prince G. A. Porter D. D. et al. (2007). Age-related differences in pulmonary cytokine response to respiratory syncytial virus infection: Modulation by anti-inflammatory and antiviral treatment. *J. Infect. Dis*. 195, 511–518. 10.1086/510628 17230410

[B6] Bruns H. Meinken C. Schauenberg P. Härter G. Kern P. Modlin R. L. et al. (2009). Anti-TNF immunotherapy reduces CD8+ T cell-mediated antimicrobial activity against *Mycobacterium* tuberculosis in humans. *J. Clin. Invest*. 119, 1167–1177. 10.1172/JCI38482 19381021 PMC2673881

[B7] Callahan B. J. McMurdie P. J. Rosen M. J. Han A. W. Johnson A. J. Holmes S. P. (2016). DADA2: High-resolution sample inference from illumina amplicon data. *Nat. Methods* 13, 581–583. 10.1038/nmeth.3869 27214047 PMC4927377

[B8] Cha S. B. Jeon B. Y. Kim W. S. Kim J. S. Kim H. M. Kwon K. W. et al. (2015). Experimental reactivation of pulmonary *Mycobacterium* avium complex infection in a modified cornell-like murine model. *PLoS One* 10:e0139251. 10.1371/journal.pone.0139251 26406237 PMC4583228

[B9] ChandraP. FaizanM. PorwalM. SharmaH. SachanN. (2026). An overview and review of growth factors in wound healing: Emerging trends and innovations. *Curr. Diabetes Rev*. 22, 1–27. 10.2174/0115733998332692241202072249 40444617

[B10] Chen F. Szymanski E. P. Olivier K. N. Liu X. Tettelin H. Holland S. M. et al. (2017). Whole-exome sequencing identifies the 6q12-q16 linkage region and a candidate gene, TTK, for pulmonary nontuberculous mycobacterial disease. *Am. J. Respir Crit. Care. Med*. 196, 1599–1604. 10.1164/rccm.201612-2479OC 28777004 PMC5754439

[B11] Chen R. Y. Zhu Y. Shen Y. Y. Xu Q. Y. Tang H. Y. Cui N. X. et al. (2023). The role of PD-1 signaling in health and immune-related diseases. *Front. Immunol*. 14:1163633. 10.3389/fimmu.2023.1163633 37261359 PMC10228652

[B12] Cinco I. R. Napier E. G. Rhoades N. S. Davies M. H. Allison D. B. Kohama S. G. et al. (2024). Immunological and microbial shifts in the aging rhesus macaque lung during nontuberculous mycobacterial infection. *mBio* 15, e0082924. 10.1128/mbio.00829-24 38771046 PMC11237422

[B13] Desai T. J. Cardoso W. V. (2002). Growth factors in lung development and disease: Friends or foe? *Respir Res*. 3:2. 10.1186/rr169 11806837 PMC64813

[B14] Dhasmana D. J. Whitaker P. van der Laan R. Frost F. (2024). A practical guide to the diagnosis and management of suspected Non-tuberculous mycobacterial pulmonary disease (NTM-PD) in the United Kingdom. *NPJ Prim. Care Respir. Med*. 34:45. 10.1038/s41533-024-00403-9 39709516 PMC11663218

[B15] Dickson R. P. Erb-Downward J. R. Freeman C. M. McCloskey L. Falkowski N. R. Huffnagle G. B. et al. (2017). Bacterial topography of the healthy human lower respiratory tract. *mBio* 8, e2216–e2287. 10.1128/mBio.02287-16 28196961 PMC5312084

[B16] Fremond C. Allie N. Dambuza I. Grivennikov S. I. Yeremeev V. Quesniaux V. F. et al. (2005). Membrane TNF confers protection to acute mycobacterial infection. *Respir Res*. 6:136. 10.1186/1465-9921-6-136 16285886 PMC1325056

[B17] Gold M. C. Cerri S. Smyk-Pearson S. Cansler M. E. Vogt T. M. Delepine J. et al. (2010). Human mucosal associated invariant T cells detect bacterially infected cells. *PLoS Biol*. 8:e1000407. 10.1371/journal.pbio.1000407 20613858 PMC2893946

[B18] Harada K. Vu Q. T. Nishimura Y. Takeda T. Hamano H. Minato Y. et al. (2025). Trends in nontuberculous mycobacterial disease mortality based on 2000-2022 data from 83 countries. *Int. J. Infect. Dis*. 158:107932. 10.1016/j.ijid.2025.107932 40354928

[B19] Haverkamp M. H. van de Vosse E. van Dissel J. T. (2014). Nontuberculous mycobacterial infections in children with inborn errors of the immune system. *J. Infect*. 68, S134–S150. 10.1016/j.jinf.2013.09.024 24119826

[B20] Hill A. R. (1991). Mycobacterial infections in AIDS. *Can. J. Infect. Dis*. 2, 19–29. 10.1155/1991/476503 22451748 PMC3307397

[B21] Honda J. R. Knight V. Chan E. D. (2015). Pathogenesis and risk factors for nontuberculous mycobacterial lung disease. *Clin. Chest. Med*. 36, 1–11. 10.1016/j.ccm.2014.10.001 25676515

[B22] Jeon D. (2019). Infection source and epidemiology of nontuberculous mycobacterial lung disease. *Tuberc. Respir Dis*. 82, 94–101. 10.4046/trd.2018.0026 30302953 PMC6435933

[B23] Katoh K. Standley D. M. (2013). MAFFT multiple sequence alignment software version 7: Improvements in performance and usability. *Mol. Biol. Evol*. 30, 772–780. 10.1093/molbev/mst010 23329690 PMC3603318

[B24] Langille M. G. Zaneveld J. Caporaso J. G. McDonald D. Knights D. Reyes J. A. et al. (2013). Predictive functional profiling of microbial communities using 16S rRNA marker gene sequences. *Nat. Biotechnol*. 31, 814–821. 10.1038/nbt.2676 23975157 PMC3819121

[B25] Lee K. A. Flores R. R. Jang I. H. Saathoff A. Robbins P. D. (2022). Immune senescence, immunosenescence and aging. *Front. Aging*. 3:900028. 10.3389/fragi.2022.900028 35821850 PMC9261375

[B26] Lee S. Y. Mac Aogáin M. Fam K. D. Chia K. L. Binte Mohamed Ali N. A. Yap M. M. C. et al. (2019). Airway microbiome composition correlates with lung function and arterial stiffness in an age-dependent manner. *PLoS One* 14:e0225636. 10.1371/journal.pone.0225636 31770392 PMC6879132

[B27] Liu B. Kimura Y. (2007). Local immune response to respiratory syncytial virus infection is diminished in senescence-accelerated mice. *J. Gen. Virol*. 88, 2552–2558. 10.1099/vir.0.83089-0 17698666

[B28] Liu Z. Liang Q. Ren Y. Guo C. Ge X. Wang L. et al. (2023). Immunosenescence: Molecular mechanisms and diseases. *Signal Transduct. Target. Ther*. 8:200. 10.1038/s41392-023-01451-2 37179335 PMC10182360

[B29] Loebinger M. R. Quint J. K. van der Laan R. Obradovic M. Chawla R. Kishore A. et al. (2023). Risk factors for nontuberculous mycobacterial pulmonary disease: A systematic literature review and meta-analysis. *Chest* 164, 1115–1124. 10.1016/j.chest.2023.06.014 37429481

[B30] Lozupone C. Lladser M. E. Knights D. Stombaugh J. Knight R. (2011). UniFrac: An effective distance metric for microbial community comparison. *ISME J*. 5, 169–172. 10.1038/ismej.2010.133 20827291 PMC3105689

[B31] Mallick H. Rahnavard A. McIver L. J. Ma S. Zhang Y. Nguyen L. H. et al. (2021). Multivariable association discovery in population-scale meta-omics studies. *PLoS Comput. Biol*. 17:e1009442. 10.1371/journal.pcbi.1009442 34784344 PMC8714082

[B32] McGarvey J. Bermudez L. E. (2002). Pathogenesis of nontuberculous mycobacteria infections. *Clin. Chest. Med*. 23, 569–583. 10.1016/s0272-5231(02)00012-6 12370993

[B33] Napier E. G. Doratt B. M. Cinco I. R. Stuart E. V. Geron R. D. Davies M. H. et al. (2025). Age-related severity of nontuberculous mycobacterial lung disease is mediated by aberrant macrophage responses and lung microbial dysbiosis. *J. Infect*. 91:106626. 10.1016/j.jinf.2025.106626 41077197 PMC12666862

[B34] Nqwata L. Ouédrago A. R. (2022). Non-tuberculous mycobacteria pulmonary disease: A review of trends, risk factors, diagnosis and management. *Afr. J. Thorac. Crit. Care Med.* 28:107196/AJTCCM2022v28i2157. 36034054 10.7196/AJTCCM.2022.v28i2.157 PMC9394508

[B35] Oh S. J. Lee J. K. Shin O. S. (2019). Aging and the immune system: The impact of immunosenescence on viral infection, immunity and vaccine immunogenicity. *Immune. Netw*. 19, e37. 10.4110/in.2019.19.e37 31921467 PMC6943173

[B36] Parada A. E. Needham D. M. Fuhrman J. A. (2016). Every base matters: Assessing small subunit rRNA primers for marine microbiomes with mock communities, time series and global field samples. *Environ. Microbiol*. 18, 1403–1414. 10.1111/1462-2920.13023 26271760

[B37] Philley J. V. Kannan A. Olusola P. McGaha P. Singh K. P. Samten B. et al. (2019). Microbiome diversity in sputum of nontuberculous mycobacteria infected women with a history of breast cancer. *Cell. Physiol. Biochem*. 52, 263–279. 10.33594/000000020 30816674

[B38] Pierre-Audigier C. Jouanguy E. Lamhamedi S. Altare F. Rauzier J. Vincent V. et al. (1997). Fatal disseminated *Mycobacterium* smegmatis infection in a child with inherited interferon gamma receptor deficiency. *Clin. Infect. Dis*. 24, 982–984. 10.1093/clinids/24.5.982 9142806

[B39] Price M. N. Dehal P. S. Arkin A. P. (2010). FastTree 2–approximately maximum-likelihood trees for large alignments. *PLoS One* 5:e9490. 10.1371/journal.pone.0009490 20224823 PMC2835736

[B40] Prudovsky I. (2021). Cellular mechanisms of FGF-stimulated tissue repair. *Cells* 10:1830. 10.3390/cells10071830 34360000 PMC8304273

[B41] Quast C. Pruesse E. Yilmaz P. Gerken J. Schweer T. Yarza P. et al. (2013). The SILVA ribosomal RNA gene database project: Improved data processing and web-based tools. *Nucleic. Acids. Res*. 41, D590–D596. 10.1093/nar/gks1219 23193283 PMC3531112

[B42] Rhoades N. S. Davies M. Lewis S. A. Cinco I. R. Kohama S. G. Bermudez L. E. et al. (2022). Functional, transcriptional, and microbial shifts associated with healthy pulmonary aging in rhesus macaques. *Cell. Rep*. 39:110725. 10.1016/j.celrep.2022.110725 35443183 PMC9096119

[B43] Ritzmann F. Lunding L. P. Bals R. Wegmann M. Beisswenger C. (2022). IL-17 cytokines and chronic lung diseases. *Cells* 11:2132. 10.3390/cells11142132 35883573 PMC9318387

[B44] Rivera A. Rais M. Barr T. Arnold N. Sureshchandra S. Messaoudi I. (2019). “Nonhuman primate models of immunosenescence,” in *Handbook of Immunosenescence*, eds Fulop T. Franceschi C. Hirokawa K. Pawelec G. (Cham: Springer Nature), 125–152.

[B45] Speranza E. Purushotham J. N. Port J. R. Schwarz B. Flagg M. Williamson B. N. et al. (2022). Age-related differences in immune dynamics during SARS-CoV-2 infection in rhesus macaques. *Life Sci. Alliance*. 5:e202101314. 10.26508/lsa.202101314 35039442 PMC8807873

[B46] Sulaiman I. Wu B. G. Li Y. Scott A. S. Malecha P. Scaglione B. et al. (2018). Evaluation of the airway microbiome in nontuberculous mycobacteria disease. *Eur. Respir. J*. 52:1800810. 10.1183/13993003.00810-2018 30093571

[B47] Sun L. He C. Nair L. Yeung J. Egwuagu C. E. (2015). Interleukin 12 (IL-12) family cytokines: Role in immune pathogenesis and treatment of CNS autoimmune disease. *Cytokine* 75, 249–255. 10.1016/j.cyto.2015.01.030 25796985 PMC4553122

[B48] Toapanta F. R. Ross T. M. (2009). Impaired immune responses in the lungs of aged mice following influenza infection. *Respir Res*. 10:112. 10.1186/1465-9921-10-112 19922665 PMC2785782

[B49] Whittington R. Marsh I. Choy E. Cousins D. (1998). Polymorphisms in IS1311, an insertion sequence common to *Mycobacterium* avium and M. Avium subsp. Paratuberculosis, can be used to distinguish between and within these species. *Mol. Cell. Probes*. 12, 349–358. 10.1006/mcpr.1998.0194 9843652

[B50] Winthrop K. L. Baxter R. Liu L. Varley C. D. Curtis J. R. Baddley J. W. et al. (2013). Mycobacterial diseases and antitumour necrosis factor therapy in USA. *Ann. Rheum. Dis*. 72, 37–42. 10.1136/annrheumdis-2011-200690 22523429

[B51] Winthrop K. L. Chang E. Yamashita S. Iademarco M. F. LoBue P. A. (2009). Nontuberculous mycobacteria infections and anti-tumor necrosis factor-alpha therapy. *Emerg. Infect. Dis*. 15, 1556–1561. 10.3201/eid1510.090310 19861045 PMC2866401

[B52] Winthrop K. L. Yamashita S. Beekmann S. E. Polgreen P. M. (2008). Mycobacterial and other serious infections in patients receiving anti-tumor necrosis factor and other newly approved biologic therapies: Case finding through the emerging infections network. *Clin. Infect. Dis*. 46, 1738–1740. 10.1086/587989 18419421

[B53] Winthrop K. Rivera A. Engelmann F. Rose S. Lewis A. Ku J. et al. (2016). A rhesus macaque model of pulmonary nontuberculous mycobacterial disease. *Am. J. Respir Cell. Mol. Biol*. 54, 170–176. 10.1165/rcmb.2015-0256RC 26562499 PMC4821046

[B54] Wong T. M. Boyapalle S. Sampayo V. Nguyen H. D. Bedi R. Kamath S. G. et al. (2014). Respiratory syncytial virus (RSV) infection in elderly mice results in altered antiviral gene expression and enhanced pathology. *PLoS One* 9:e88764. 10.1371/journal.pone.0088764 24558422 PMC3928298

[B55] Wrona M. V. Ghosh R. Coll K. Chun C. Yousefzadeh M. J. (2024). The 3 i’s of immunity and aging: Immunosenescence, inflammaging, and immune resilience. *Front. Aging*. 5:1490302. 10.3389/fragi.2024.1490302 39478807 PMC11521913

[B56] Wu U. I. Holland S. M. (2015). Host susceptibility to non-tuberculous mycobacterial infections. *Lancet Infect. Dis*. 15, 968–980. 10.1016/S1473-3099(15)00089-4 26049967

[B57] Yangco B. G. Lackman-Smith C. Espinoza C. G. Solomon D. A. Deresinski S. C. (1989). The hamster model of chronic *Mycobacterium* avium complex infection. *J. Infect. Dis*. 159, 556–561. 10.1093/infdis/159.3.556 2644384

[B58] Zeng G. Zhang G. Chen X. (2018). Th1 cytokines, true functional signatures for protective immunity against TB? *Cell. Mol. Immunol*. 15, 206–215. 10.1038/cmi.2017.113 29151578 PMC5843617

