# Supplementary material for: Lung microbial dysregulation and TNF inhibition are associated with worsened nontuberculous mycobacterial lung disease
Source: Front Microbiol. 2026 Sep 15;17:1923035. doi: 10.3389/fmicb.2026.1923035 (PMC13619843; doi:10.3389/fmicb.2026.1923035)
Supplement: Supplementary file 1 [file Table_1.docx]

| **Table S1: Metadata of the research animals investigated during this study** | | | | | | |
| --- | --- | --- | --- | --- | --- | --- |
| Blinded ID | DOB | Sex | Inoculation date | Weight at Inoculation | Age at Inoculation (years) | Group |
| C1 | 5/17/17 | F | 11/29/23 | 5.10 | 6.4 | Control |
| C2 | 4/10/18 | M | 11/29/23 | 8.00 | 5.5 | Control |
| C3 | 7/17/18 | F | 11/29/23 | 5.25 | 5.2 | Control |
| C4 | 5/10/19 | M | 11/29/23 | 8.55 | 4.4 | Control |
| A1 | 6/6/17 | F | 11/29/23 | 5.30 | 6.3 | Antibiotics |
| A2 | 5/4/19 | M | 11/29/23 | 7.70 | 4.4 | Antibiotics |
| A3 | 2/12/19 | F | 11/29/23 | 5.30 | 4.6 | Antibiotics |
| A4 | 3/22/19 | M | 11/29/23 | 9.15 | 5.2 | Antibiotics |
| I1 | 3/19/19 | M | 11/29/23 | 9.10 | 4.5 | Inflectra |
| I2 | 4/23/19 | F | 11/29/23 | 4.90 | 4.4 | Inflectra |
| I3 | 5/18/18 | F | 11/29/23 | 7.85 | 5.4 | Inflectra |
| I4 | 5/19/19 | M | 11/29/23 | 6.60 | 4.4 | Inflectra |
